# Erbium oxide nanoparticles induce potent cell death, genomic instability and ROS-mitochondrial dysfunction-mediated apoptosis in U937 lymphoma cells

**DOI:** 10.1007/s00210-025-03962-x

**Published:** 2025-03-12

**Authors:** Hanan R. H. Mohamed, Yusuf Ahmed Elberry, Hagar Magdy, Maryam Ismail, Maivel Michael, Nourhan Eltayeb, Gehan Safwat

**Affiliations:** 1 Department of Zoology, Faculty of Science, Cairo University, Giza, Egypt; 2 Faculty of Biotechnology, October University for Modern Sciences and Arts (MSA), 6th of October City, Egypt

**Keywords:** Erbium oxide nanoparticles, U937 lymphoma cells, WST-1 assay, ROS generation, Genomic instability and apoptosis induction

## Abstract

Erbium oxide nanoparticles (Er_2_O_3_-NPs) have attracted significant attention for their unique physicochemical properties, including high surface area, biocompatibility, and stability. However, the impact of Er_2_O_3_-NPs on lymphoma cells (LCs) has not been explored, making this an innovative avenue for exploration. Therefore, the current study aimed to explore the influence of Er_2_O_3_-NPs on cell viability, genomic and mitochondrial DNA integrity, reactive oxygen species (ROS) generation and apoptosis induction in human U937 LCs. Er_2_O_3_-NPs were characterized using X-ray diffraction (XRD) and transmission electron microscopy (TEM). The effect of Er_2_O_3_-NPs on cell viability and genomic DNA integrity was estimated after 48 h using the WST-1 cytotoxicity and alkaline Comet assays, respectively. The generation level of reactive oxygen species (ROS) and mitochondrial membrane potential were also analyzed. Flow Cytometry was used to assess apoptosis induction and quantitative RT-PCR was conducted to measure the apoptotic (*p53*), anti-apoptotic (*Bcl2*), and mitochondrial (*ND3*) gene expression. Our results demonstrated the purity and well distribution of Er_2_O_3_-NPs and revealed that Er_2_O_3_-NPs induce strong cytotoxicity on U937 cells, as evidenced by a concentration-dependent reduction in cell viability with an IC50 value of 3.20 µg/ml. Exposure of U937 LCs to the IC50 concentration (3.20 µg/ml) of Er_2_O_3_-NPs promoted excessive ROS generation, leading to dramatic damage to genomic DNA and mitochondrial membrane potential, as well as marked dysregulation of apoptotic (*p53*), anti-apoptotic (*Bcl2*) and mitochondrial *ND3* gene expression. This cascade of events triggered both apoptosis and necrosis in Er_2_O_3_-NPs-treated U937 LCs. In conclusion, these findings highlight the strong in vitro cytotoxic potential of Er_2_O_3_-NPs against highly aggressive U937 LCs, mediated by excessive ROS production, which leads to severe genomic DNA and mitochondrial membrane damage, as well as profound alterations in apoptotic, anti-apoptotic and mitochondrial gene expression. Future research is needed to further investigate the potential use of Er_2_O_3_-NPs in treating lymphoma and to optimize their therapeutic efficacy.

## Introduction

Lymphoma, a diverse group of hematologic malignancies originating in the lymphatic system, poses a significant global health challenge due to its complexity and unpredictable clinical outcomes. The incidence of lymphoma has been steadily rising worldwide, with two primary subtypes: non-Hodgkin lymphoma and Hodgkin lymphoma, each exhibiting distinct epidemiological and clinical features. Non-Hodgkin lymphoma accounts for the majority of cases, with risk factors including immunosuppression, viral infections, genetic predisposition, and environmental exposures (Sung et al. 2021; Shankland et al. 2012). Although less common, Hodgkin lymphoma primarily affects young adults and is often associated with immune dysfunction and viral infections (Wang et al. 2019; Velasco-Suelto et al. 2024).

Lymphoma's distribution is influenced by a range of factors, including geographic, genetic, and socioeconomic determinants. Higher rates are seen in developed regions, which may be attributed to advanced diagnostic techniques and greater exposure to risk factors (Bispo et al. 2020; Wang et al. 2024). Despite these disparities, lymphoma remains a significant contributor to cancer-related morbidity and mortality worldwide.

Over recent years, significant advances in lymphoma treatment have been made, including chemotherapy, immunotherapy, radiotherapy, and targeted therapies. Chemotherapy remains a cornerstone of lymphoma management, with regimens such as ABVD (doxorubicin, bleomycin, vinblastine, dacarbazine) for Hodgkin lymphoma and CHOP (cyclophosphamide, doxorubicin, vincristine, prednisone) for non-Hodgkin lymphoma demonstrating high efficacy in achieving remission (Evens et al. 2008; Ansell 2015; Che et al. 2023). However, despite its effectiveness, chemotherapy can cause significant side effects that negatively impact patients' quality of life and long-term health outcomes.

These side effects stem from chemotherapy's non-specific mechanism of action, which targets rapidly dividing cells and impacts both malignant and healthy tissues. Common acute toxicities include nausea, vomiting, myelosuppression, and mucositis. Long-term complications such as cardiotoxicity from anthracyclines and pulmonary fibrosis from bleomycin pose serious risks, particularly for younger patients with curable disease (Anand et al. 2022; Camilli et al. 2024). Additionally, chemotherapy increases the likelihood of secondary malignancies and infertility, raising concerns about long-term survivorship (Poorvu et al. 2019; Nikkilä et al. 2024).

The risk of these adverse effects varies based on treatment intensity, patient age, comorbidities, and cumulative drug doses. Although supportive care measures, such as anti-emetics and growth factors, help alleviate some side effects, the long-term toxicities underscore the need for personalized treatment strategies (Ansell 2015). Exploring alternative therapies is thus essential to minimize toxicity. Nanotherapy has emerged as a promising cancer treatment strategy by leveraging nanoparticles to enhance drug delivery, reduce systemic toxicity, and improve therapeutic efficacy. In lymphoma treatment, nanotechnology has garnered attention for developing targeted therapies that overcome the limitations of conventional chemotherapy, such as non-specificity and severe side effects (Cheng et al. 2023; Mohamed et al. 2025a).

Nanoparticles, particularly erbium oxide nanoparticles (Er_2_O_3_ NPs) show great potential due to their unique physicochemical properties, including high thermal stability, photoluminescence, and biocompatibility (Safwat et al. 2022; Mohamed et al. 2023a). These properties make them ideal for imaging and theranostic applications, enabling simultaneous cancer detection and treatment (Sau et al. 2018). Understanding how Er_2_O_3_ NPs interact with LCs is crucial for advancing treatment strategies.

Unfortunately, there is a notable lack of data on the cytotoxicity and genotoxicity of Er_2_O_3_ NPs in both normal and cancer cells. Recent studies on human skin fibroblasts (HSF) and hepatocellular carcinoma (Hep-G2) cells revealed that exposure to an IC50 concentration (6.21 µg/ml) of Er_2_O_3_ NPs for 72 h induced oxidative stress in Hep-G2 cells. This resulted in overproduction of reactive oxygen species (ROS), disrupting cellular homeostasis and genomic stability, leading to cell cycle arrest at the G0/G1 phase and subsequent apoptosis and necrosis. In contrast, normal HSF cells showed no significant changes in ROS levels, genomic DNA integrity, or apoptotic gene expression after similar exposure (Safwat et al. 2022; Mohamed et al. 2023b). The excessive ROS generation appears to be a key mechanism by which Er_2_O_3_ NPs exert their anticancer effects, making them particularly effective against cancer cells that resist conventional therapies (Safwat et al. 2022).

Given the promising anticancer effect of Er_2_O_3_ NPs against hepatic cancer Hep-G2 cells (Safwat et al. 2022), alongside with the lack of data on Er_2_O_3_ NPs impact on other human cancer cell lines, particularly LCs, highlight the need to explore their effect on lymphoma. Therefore, the current study was undertaken to estimate the effect of Er_2_O_3_ NPs on the viability of human normal oral epithelial cells (OEC) and U937 LCs, as well as to evaluate genomic DNA integrity, mitochondrial membrane potential, ROS generation, and the potential induction of apoptosis induction in U937 LCs.

## Materials and methods

### Chemicals

The Er_2_O_3_ NPs used in this study were purchased from Sigma-Aldrich Chemical Company (St. Louis, MO, USA). They were supplied as a faint pink powder with an average particle size of ≤ 100 nm. These Er_2_O_3_-NPs, with a purity of 99.9% (trace metals basis), have the CAS number 12061–16-4 and EC number 235–045-7, and the product number is 203,238. All other chemicals such as Rhodamine-123 and 2,7-dichlorofluorescin diacetate (DCFH-DA) used in the experimental procedures were purchased from Sigma-Aldrich Chemical Company (St. Louis, MO, USA) with high molecular grade to ensure consistency and accuracy throughout the study.

### Characterization of Er_2_O_3_ NPs

The Er_2_O_3_ were characterized using X-ray diffraction (XRD) to confirm the purity of the purchased nano powders. The XRD patterns were obtained using a charge-coupled device diffractometer (XPERT-PRO, PANalytical, Almelo, Netherlands). The average particle size and morphology of the suspended Er_2_O_3_ NPs were also analyzed using transmission electron microscopy (TEM) with a Tecnai G20 Super Twin double-tilt TEM, operating at an accelerating voltage of 200 kV.

### Cell culture

Human U937 lymphoma cells (LCs) were obtained from Nawah Scientific Inc. (Mokatam, Cairo, Egypt). Both OEC and U937 cells were cultured separately in Dulbecco's Modified Eagle Medium (DMEM) medium (Thermo Fisher Scientific, USA) supplemented with 10% inactivated fetal bovine serum (Thermo Fisher Scientific, USA), 100 units/ml penicillin (Sigma-Aldrich, USA), and 100 mg/mL streptomycin (Sigma-Aldrich, USA). The OEC and U937 cells were maintained in an incubator set at 37°C with 5% CO_2_ to provide optimal growth conditions.

### Evaluation of Er_2_O_3_-NPs cytotoxicity

The cytotoxicity of Er_2_O_3_-NPs was estimated using the water-soluble tetrazolium salt (WST-1) assay (Sharma et al. 2014; Alaufi et al. 2017; Singh et al. 2022). This assay was conducted to measure the viability of OEC and U937 cells upon exposure to Er_2_O_3_-NPs using Abcam® kit (ab155902 WST-1 Cell Proliferation Reagent). Aliquots of 50 µl cell suspension (3 × 10^3^ cells) of OEC or U937 cells were seeded in 96-well plates and incubated in complete media for 24 h. The cells were then treated with 50 µl of media containing Er_2_O_3_-NPs at serial concentrations of 0.1, 1, 10, 100 and 1000 µg/ml. After 48 h of exposure to Er_2_O_3_-NPs, 10 µl of WST-1 reagent was added, and the absorbance was measured after 1 h at 450 nm using an Infinite F50 microplate reader (TECAN, Switzerland). The half-maximal inhibitory concentration (IC50) of Er_2_O_3_-NPs for normal OEC and U937 LCs was calculated from three replicates using GraphPad Prism software.

### Treatment schedule

U937 LCs were plated in T25 flasks under appropriate conditions and then divided into untreated (control) and treated groups. The control cells were exposed to DMSO at a concentration of less than 0.1%, while the treated U937 cells were exposed to Er_2_O_3_-NPs at a concentration equal to the IC50 value for 48 h. Following treatment, both the control and treated cells were centrifuged, trypsinized, and washed twice with ice-cold Phosphate-Buffered Saline (PBS). The harvested cells were stored at −80 °C in PBS for future molecular analysis. Triplicates of both control and treated cells were performed to ensure accuracy and consistency of the results.

### Measurement of genomic DNA damage

Genomic DNA damage in both untreated (control) and Er_2_O_3_-NPs-treated U937 cells was measured using the alkaline comet assay (Tice et al. 2000; Langie et al. 2015). A 15 µl suspension of U937 cells was mixed with 60 µl of low melting agarose, spread onto a clean slide pre-covered with normal-melting agarose (1%) and left for gel hardening. The slides were then immersed in a cold lysis buffer with freshly added DMSO and Triton-X100 and incubated in darkness at 4 °C for 24 h. After lysis, the slides were placed in freshly prepared alkaline electrophoresis buffer with pH greater than 12 for 15 min and electrophoresed at 25 V and 300 mA for 30 min. The slides were then neutralized, fixed, dried, and stained with ethidium bromide for imaging. The r comet nuclei, showing various levels of DNA damage, were analyzed using COMETSCORE™ software. Comet parameters including: tail length, %DNA in the tail, and tail moment were reported as mean ± standard deviation (SD).

### Estimation of ROS generation level

The level of ROS generation in untreated and Er_2_O_3_-NPs-treated U937 LCs was screened using 2, 7-DCFH-DA dye (Siddiqui et al. 2010). U937 cell suspension was mixed with an equal volume of 20 mM DCFH-DA dye, gently shaken, and incubated in the dark at room temperature for 30 min. During the incubation, the DCFH-DA dye enters the cells and specifically reacts with ROS, forming the fluorescent compound dichlorofluorescein (DCF). After incubation, the cell-dye mixture was spread into a thin film on a clean slide and examined under an epi-fluorescent microscope at 200X magnification. Images were captured to detect the emitted fluorescent light, which indicates ROS production in the U937 LCs. The intensity of the emitted fluorescence was quantified using Image Analysis Fiji: ImageJ software, providing a measure of ROS generation in both untreated and treated U937 cells.

### Assessing the mitochondrial membrane potential integrity

The integrity of mitochondrial membrane potential in untreated and Er_2_O_3_-NPs-treated U937 LCs was assessed using the fluorescent Rhodamine-123 dye (Zhang et al. 2011). U937 cell suspension was added to an equal volume of Rhodamine-123 fluorescent dye (10 mg/ml), gently mixed, and left in darkness for 1 h at 37°C. After incubation, the cells were washed twice with PBS, spread onto a clean sterile slide in a thin film, and examined under an epi-fluorescence microscope at 200 × magnification to detect the fluorescence emitted from the Rhodamine-123-stained cells. The intensity of the emitted fluorescence was quantified using Image Analysis Fiji: ImageJ software, providing a measure of mitochondrial membrane potential in both untreated and treated U937 cells.

### Apoptosis detection using flow cytometry

The potential for apoptosis induction in untreated and Er_2_O_3_-NPs-treated U937 LCs was estimated using Flow Cytometry, based on the manufacturer's protocol for the Annexin V- Fluorescein isothiocyanate (FITC) apoptosis detection kit (Abcam Inc., Cambridge, UK). A Flow Cytometer with dual-channel was utilized to distinguish between apoptotic and necrotic cells. After exposing U937 cell to the IC50 concentration of Er_2_O_3_-NPs for 48 h, the cells were harvested via trypsinization and washed twice with ice-cold PBS (pH 7.4). The cells were then left in darkness with an Annexin V-FITC/propidium iodide (PI) solution for 30 min at room temperature. Following incubation, cells were analyzed using the ACEA Novocyte flow cytometer (ACEA Biosciences Inc., San Diego, CA, USA), with FITC and PI fluorescence measured by FL1 and FL2 detectors, respectively (λex/em 488/530 nm for FITC and λex/em 535/617 nm for PI). A total of 12,000 events per sample were acquired, and positive FITC and/or PI cells were quantified using quadrant analysis with ACEA NovoExpress software (ACEA Biosciences Inc., San Diego, CA, USA).

### Quantification of the apoptotic and mitochondrial gene expression

Quantitative Real-Time polymerase chain reaction (qRT-PCR) was performed to measure the expression of apoptotic: *p53* and *Bcl2* and mitochondrial *ND3* genes in untreated and Er_2_O_3_-NPs-treated U937 LCs. Cellular RNA was extracted from U937 cells based on the manufacturer's instructions of Thermo Fisher Scientific's GeneJET RNA Purification Kit (USA). One µg of the extracted RNA was then reversely transcribed into complementary DNA (cDNA) using the cDNA Reverse Transcription Kit from Applied Biosystems (Foster City, CA, USA). A qRT-PCR was carried out for each gene using SYBR Green PCR Master Mix and the primer sequences provided in Table 1 (Suzuki et al. 1999; Lai et al. 2013; Grzybowska-Szatkowska and Ślaska 2014) on the StepOnePlus Real-Time PCR System (Applied Biosystems). The expression level of the *p53, ND3* and *Bcl2* genes were calibrated against GAPDH (housekeeping gene) expression and the comparative Ct (ΔΔCt) method was utlized to calculate the fold change in the expression of tested genes. Results are presented as mean ± SD.

**Table 1:** Sequences of primers used in qRT-PCR

Gene	Strand	Primer's sequences
GAPDH	Forward	5'-GAAGGTGAAGGTCGGAGTCA-3'
	Reverse	5'-GAAGATGGTGATGGGATTTC-3'
ND3	Forward	5'-CGCCGCCTGATACTGGCAT-3’
	Reverse	5'-CTAGTATTCCTAGAAGTGAG-3'
BCL-2	Forward	5'-TCCGATCAGGAAGGCTAGAGT-3'
	Reverse	5'-TCGGTCTCCTAAAAGCAGGC-3'
P53	Forward	5'-CAGCCAAGTCTGTGACTTGCACGTAC-3'
	Reverse	5'-CTATGTCGAAAAGTGTTTCTGTCATC-3'

### Statistical analysis

The results of this study were analyzed using the Statistical Package for the Social Sciences (SPSS) and are presented as mean ± SD. An unpaired Student's t-test was applied to compare the treated and untreated cells at *p* < 0.05 level.

## Results

### Characterization of Er_2_O_3_-NPs

**Fig. 1:**
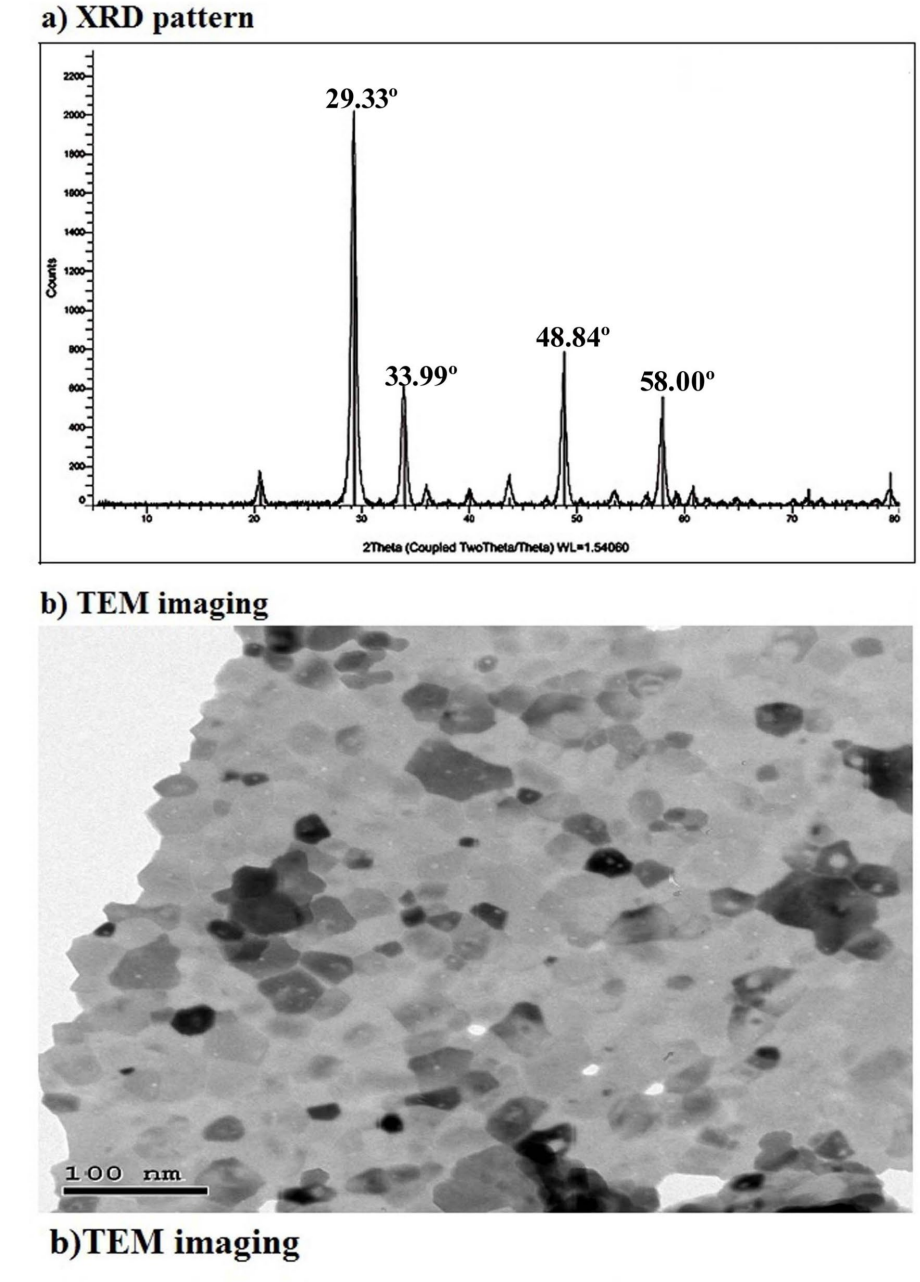
Characterization profile of Er_2_O_3_ NPs showing (**a**) X-Ray Diffraction (XRD) pattern and (**b**) Transmission electron microscopy (TEM) imaging

XRD characterization of Er_2_O_3_-NPs revealed their crystallographic properties through distinct peaks corresponding to specific crystal planes of Er_2_O_3_-NPs. These diffraction peaks, observed at angles 29.33º, 33.99º, 48.84º, and 58.00º as seen in Fig. 1, align with known reference patterns, confirming the formation of cubic and spherical phases of Er_2_O_3_-NPs. TEM imaging further corroborated this, showing the cubic and spherical morphology of the suspended Er_2_O_3_-NPs. The TEM images also demonstrated a well-distributed suspension of Er_2_O_3_-NPs in the aqueous medium, with an average particle size of 60.67 ± 4.43 nm as displayed in Fig. 1.

### Strong cytotoxicity of Er_2_O_3_-NPs against U937 cells

**Fig. 2:**
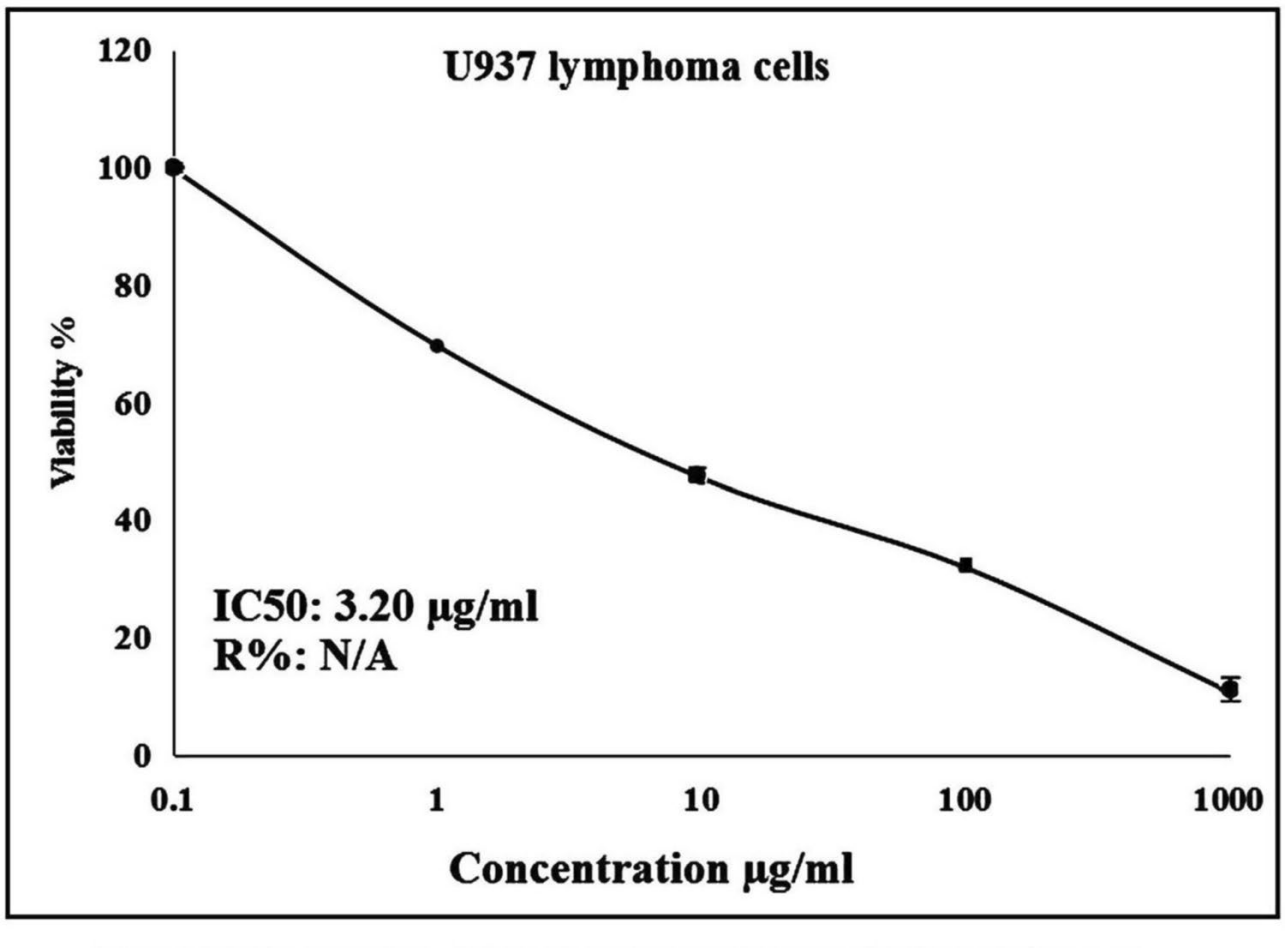
Viability profile of human U937 LCs after 48 h of exposure to Er_2_O_3_ NPs at five concentrations (0.1, 1, 10, 100, and 1000 µg/ml), assessed using the WST-1 assay

The results of the WST-1 cytotoxicity assay demonstrated a strong cytotoxicity of Er_2_O_3_-NPs against the highly aggressive U937 lymphoma cancer cells, as displayed in Fig. 2. A dramatic reduction in U937 cell viability was noticed after 48 h of exposure to Er_2_O_3_-NPs at concentrations of 0.1, 1, 10, 100 and 1000 µg/ml, in a concentration-dependent manner, with an IC50 value of 3.20 µg /ml as seen in Fig. 2.

### Er_2_O_3_-NPs induce dramatic genomic DNA damage within U937 cells

**Fig. 3:**
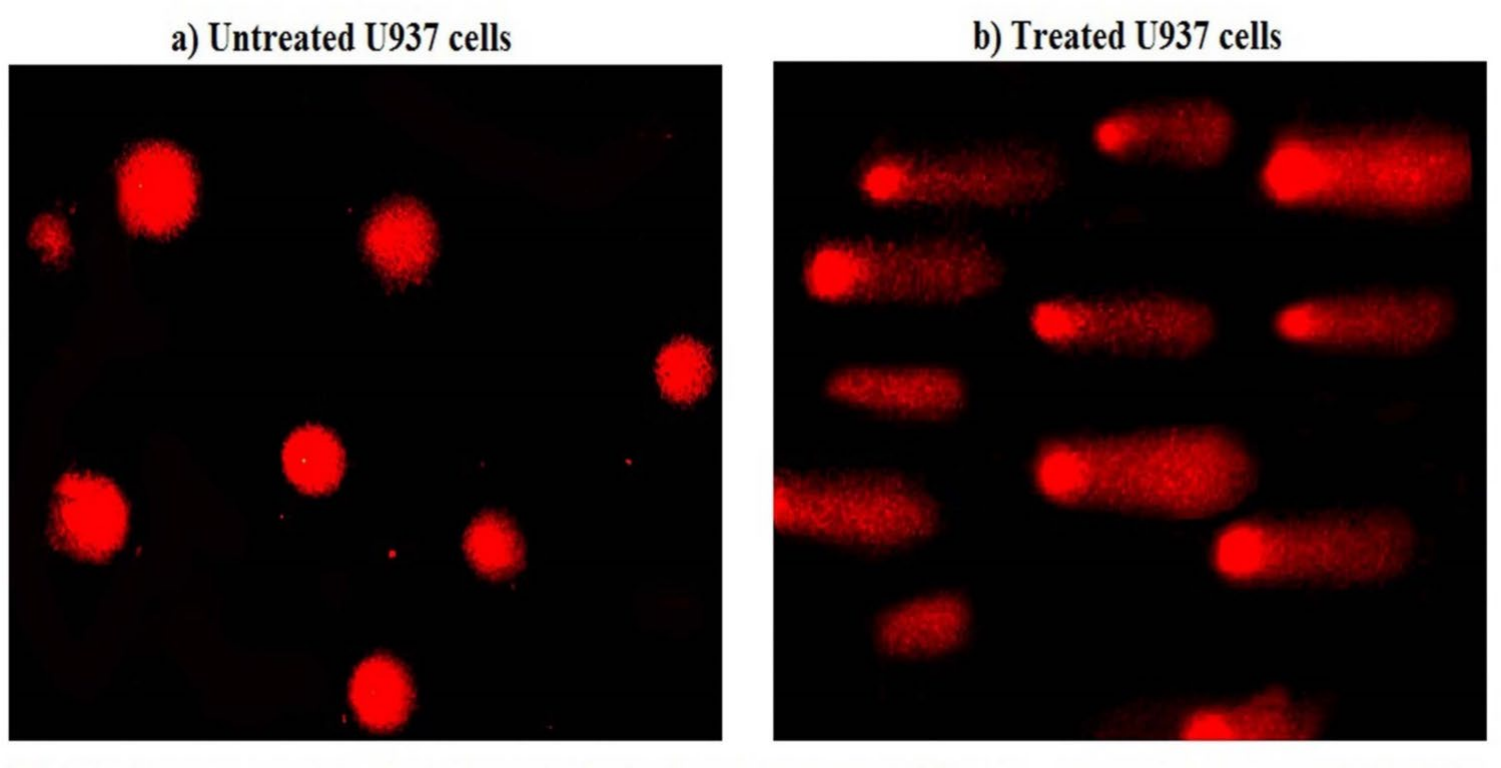
Comet nuclei with intact DNA in (**a**) untreated U937 cells and with damaged DNA in (**b**) U937 cells treated with the IC50 concentration of Er_2_O_3_ NPs for 48 h. Magnification 200x

Measurement of the DNA damage using the alkaline Comet assay revealed that exposure of U937 LCs to Er_2_O_3_-NPs at the IC50 concentration (3.20 µg/ml) for 48 h caused dramatic damage in the genomic DNA as displayed in Table 2 and Fig. 3. This damage was manifested by the statistical significant increases in key DNA damage parameters: tail length (*p* < 0.001), %DNA in tail (*p* < 0.01) and tail moment (*p* < 0.001) in Er_2_O_3_-NPs-treated U937 LCs compared to their values in untreated U937 cells as shown in Table 2. Examples for the scored Comet nuclei with intact and damaged DNA are shown in Fig. 3.

**Table 2:** Induction of genomic DNA damage in the untreated and treated Human Hystocytic lymphoma (U937) cells with IC50 concentration of Er_2_O_3_-NPs

Cells	Treatment (µg/ml)	Tail length (px)	%DNA in tail	Tail moment
U937 LCs	Er_2_O_3_-NPs (0.00)	2.79 ± 0.58	13.62 ± 1.50	0.39 ± 0.09
	Er_2_O_3_-NPs (3.20)	11.20 ± 0.95 ^***^	31.38 ± 1.74 ^**^	3.49 ± 0.14 ^***^

Results are expressed as mean ± SD

**^,^ ***Indicates statistical significant difference from the compared untreated control cells at *p* < 0.01 and *p* < 0.001, respectively, using *independent student t-test*

### Er_2_O_3_-NPs over generate ROS within U937 cells

**Fig. 4:**
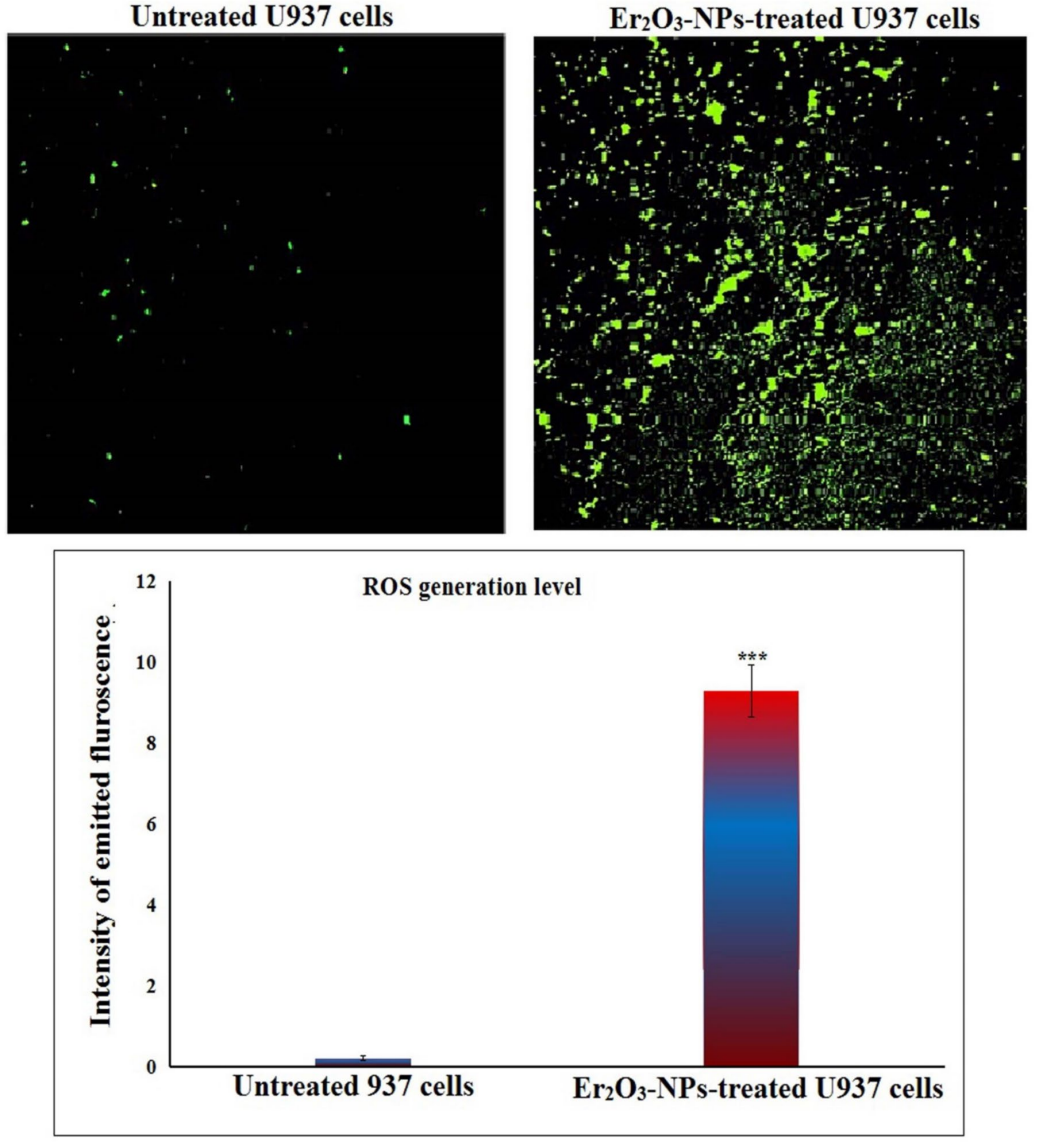
Level of ROS generation within the untreated and treated U937 cells with the IC50 concentration of Er_2_O_3_ NPs for 48 h. Magnification 200x. ***: Indicates statistical significant difference from the compared untreated control cells at *p* < 0.001 using *independent student t-test*

Screening ROS generation within the untreated and Er_2_O_3_-NPs-treated U937 LCs using 2,7- DCFH-DA dye demonstrated a significant over production of ROS within U937 LCs after 48 h of exposure to Er_2_O_3_-NPs. This was evidenced by a marked increase in the intensity of fluorescence emitted by the Er_2_O_3_-NPs-treated U937 LCs compared to the fluorescence from the untreated U937 cells as displayed in Fig. 4.

### Er_2_O_3_-NPs dramatically disrupt U937 cells mitochondrial membrane integrity

**Fig. 5:**
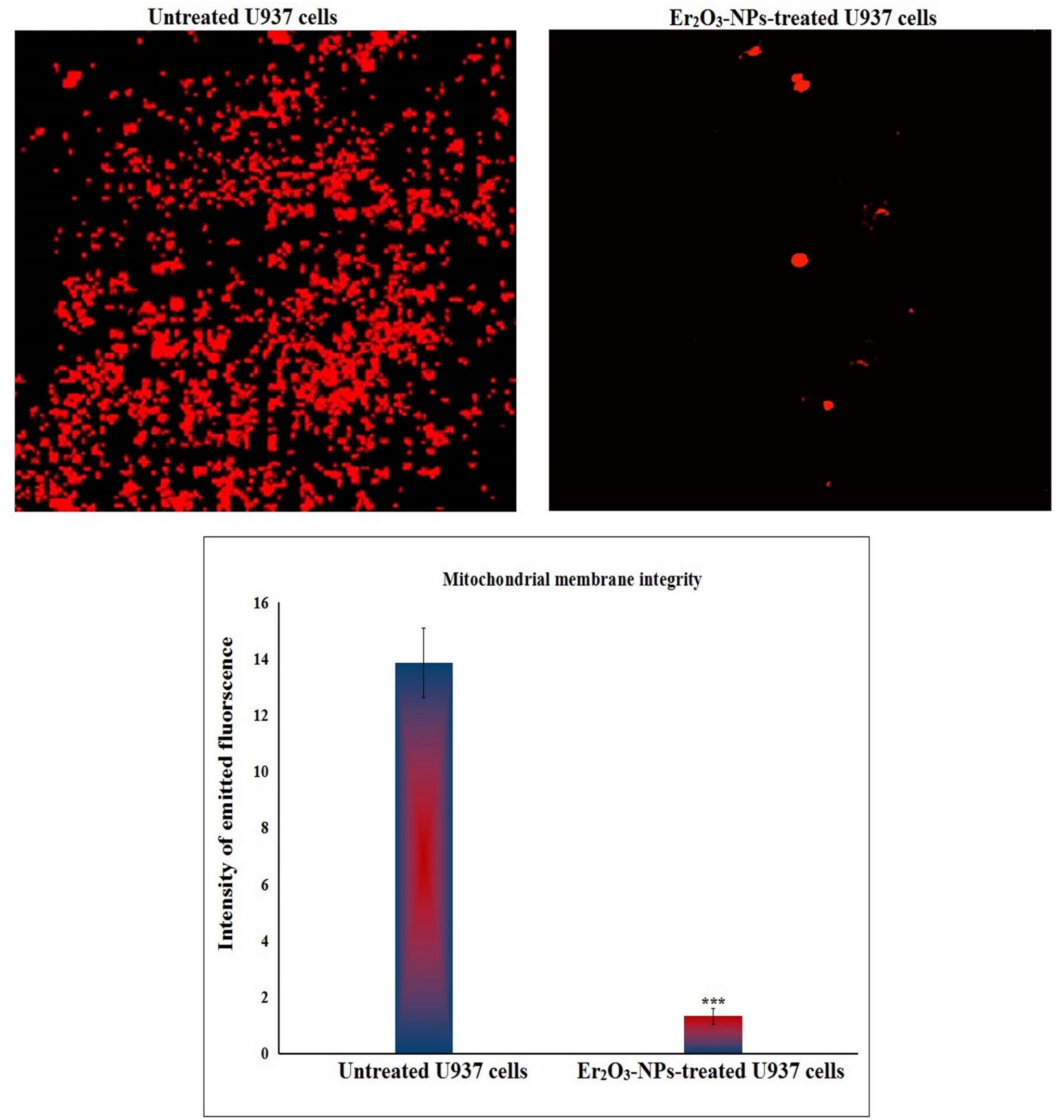
Integrity of mitochondrial membrane potential in the untreated and treated U937 cells with the IC50 concentration of Er_2_O_3_ NPs for 48 h. Magnification 200x. ***: Indicates statistical significant difference from the compared untreated control cells at *p* < 0.001 using *independent student t-test*

Assessment of the integrity of mitochondrial membrane potential using the Rhodamine-123 fluorescent dye revealed a dramatic damage to the mitochondrial membrane potential in the U937 LCs treated with Er_2_O_3_-NPs as displayed in Fig. 5. This extensive damage was reflected in the notable decrease in fluorescence intensity emitted by Er_2_O_3_-NPs-treated U937 LCs, compared to the fluorescence observed in the untreated cells compared to the fluorescence as seen in Fig. 5.

### Er_2_O_3_-NPs stimulate marked apoptosis and necrosis in U937 cells

**Fig. 6:**
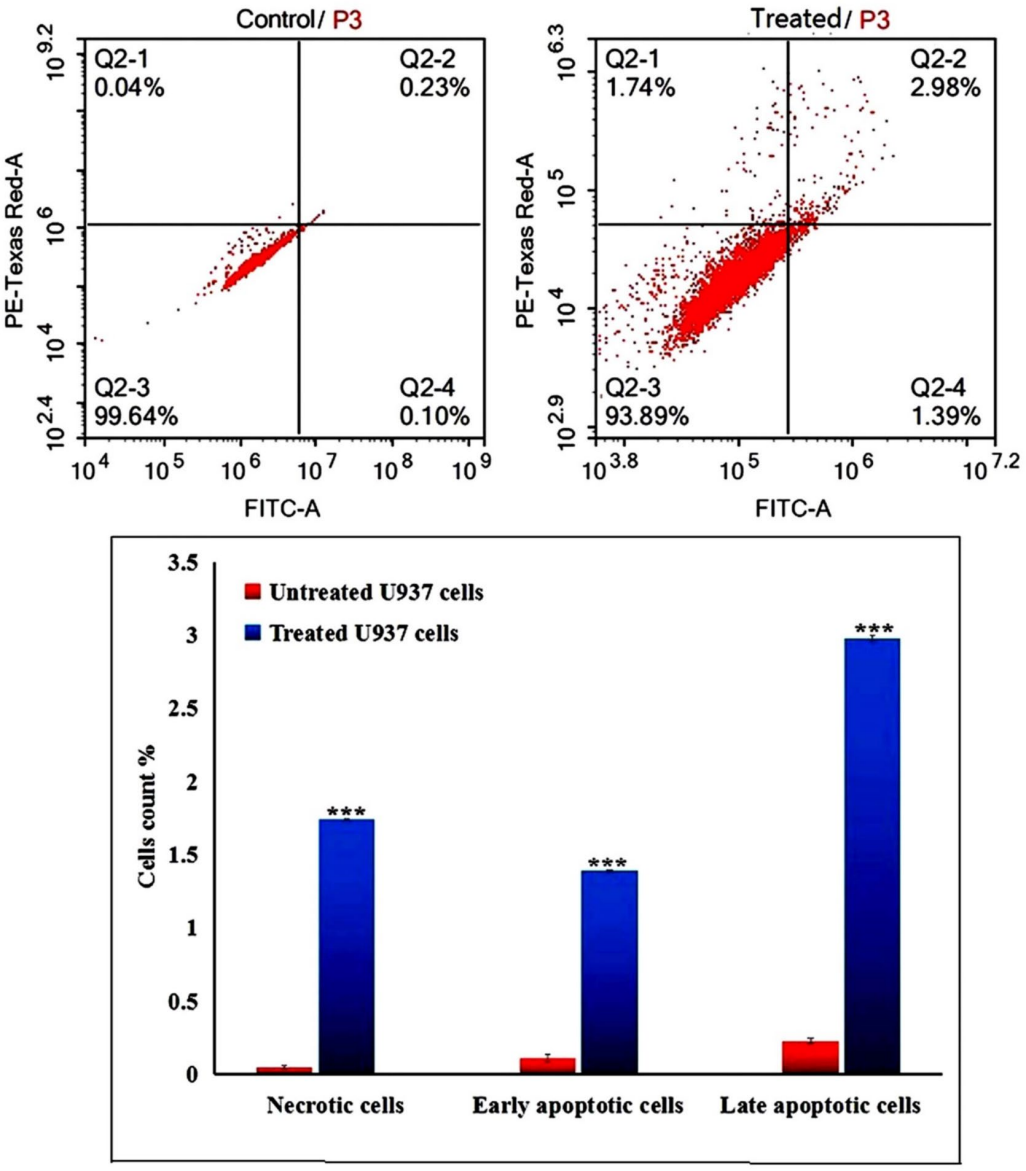
Apoptosis and necrosis induction in the untreated and treated U937 cells with the IC50 concentration of Er_2_O_3_ NPs for 48 h. Q2-1 denotes necrosis phase; Q2-2 denotes late apoptosis phase, Q2-3 denotes normal viable cells and Q2-4 denotes early apoptosis phase. ***: Indicates statistical significant difference from the compared untreated control cells at *p* < 0.001 using *independent student t-test*

Flow cytometry results demonstrated that exposure to the IC50 concentration (3.20 µg/ml) of Er_2_O_3_-NPs for 48 h led to significant apoptosis and necrosis in U937 LCs, as shown in Fig. 6. This was evidenced by statistically significant (*p* < 0.001) increases in the number of Er_2_O_3_-NPs-treated U937 cells in in both early and late apoptotic phases, as well as in the necrotic phase, compared to the untreated U937 cells in the same phases.

### Er_2_O_3_-NPs strongly disrupt apoptotic and mitochondrial gene expression in U937 cells

Quantification of the expression level of apoptosis-related genes (*p53* and *Bcl2*) and the mitochondrial *ND3* gene using qRT-PCR analysis in U937 LCs revealed that treatment with the IC50 concentration (3.20 µg/ml) of Er_2_O_3_-NPs for 48 h significantly (*p* < 0.001) downregulated the expression of the apoptotic *p53* and mitochondrial *ND3* genes, and also significantly (*p* < 0.001) upregulated the expression of the anti-apoptotic *Bcl2* gene in Er_2_O_3_-NPs -treated U937 cells compared to untreated U937 cells as shown in Table 3.

**Table 3:** Expression level of p53, ND3 and Bcl2 genes in the untreated and treated Human Hystocytic lymphoma (U937) cells with IC50 concentration of Er_2_O_3_-NPs

Cells	Treatment (µg/ml)	Fold change in the expression level of		
		p53 gene	ND3 gene	Bcl2 gene
U937 LCs	Er_2_O_3_-NPs (0.00)	1.00 ± 0.00	1.00 ± 0.00	1.00 ± 0.00
	Er_2_O_3_-NPs (3.20)	0.54 ± 0.03^***^	0.49 ± 0.01 ^***^	2.26 ± 0.07 ^***^

Results are expressed as mean ± SD

^***^Indicates statistical significant difference from the compared untreated control cells at *p* < 0.001 using *independent student t-test*

## Discussion

Nanoparticles, particularly metal oxide nanoparticles, have garnered significant attention for their potential to selectively target cancer cells. While many studies have been conducted on the cytotoxic effects of commonly used nanoparticles such as titanium dioxide, silver, and gold, the investigation of Er_2_O_3_-NPs, particularly in the context of human lymphoma, remains unexplored (Safwat et al. 2022; Al-Samydai et al. 2024; Yassin et al. 2024; Sidhic et al. 2025). To address this gap, the present study was designed to assess the impact of Er_2_O_3_-NPs exposure on cell viability, genomic and mitochondrial DNA integrity, ROS generation, and apoptosis induction in human U937 LCs.

In the context of cytotoxicity, the results of the WST-1 assay demonstrated the strong cytotoxic effect of Er_2_O_3_-NPs on the highly aggressive U937 LCs, as manifested by a remarkable concentration-dependent reduction in the viability of U937 lymphoma cell upon exposure to Er_2_O_3_-NPs for 48 h. The observed IC50 value of 3.20 µg /ml highlights the potency of these nanoparticles. This substantial reduction in U937 cell viability aligns with recent findings by Safwat et al*.* (Safwat et al. 2022), which demonstrated that Er_2_O_3_-NPs induce potent cytotoxicity in hepatocellular carcinoma Hep-G2 cells.

The cytotoxicity of nanoparticles is commonly attributed to their ability to generate excessive ROS, which damage cellular components including DNA, and disrupt cellular homeostasis (Mohamed et al. 2023c, 2024, 2025b). In this study, the significant increase in fluorescence intensity emitted by 2,7- DCFH-DA stained lymphoma ells confirmed a notable rise in ROS production following exposure to Er_2_O_3_-NPs. This ROS generation plays a central role in inducing oxidative stress, which in turn disrupts cellular functions. These findings are consistent with those of Safwat et al. (Safwat et al. 2022), who also observed excessive ROS generation in Hep-G2 hepatic cancer cells treated with the IC50 concentration of Er_2_O_3_-NPs.

The substantial increase in ROS production noticed in Er_2_O_3_-NPs -treated U937 cells is a key finding of this study. ROS are highly reactive molecules that can inflict widespread damage to lipids, proteins, and nucleic acids, leading to cellular dysfunction and death (Afzal et al. 2023; Kumar et al. 2024a, b). The elevated ROS levels detected here likely play a crucial role in inducing apoptosis, as ROS are known activators of both intrinsic and extrinsic apoptotic pathways (Rauf et al. 2024). This was further supported by our Flow Cytometry results, which revealed a significant increase in both early and late apoptotic cells, as well as necrotic cells following Er2O3-NP treatment. These results suggest that ROS-mediated apoptosis is a primary mechanism driving cell death in U937 LCs.

Cells were categorized into apoptotic, necrotic, and viable populations using a combination of Annexin V and propidium iodide (PI) staining followed by flow cytometric analysis. Viable cells are both Annexin V-negative and PI-negative, meaning they do not display external markers of apoptosis or necrosis. These cells do not bind to Annexin V (which detects phosphatidylserine translocation, an early marker of apoptosis) and are negative for PI staining (which indicates membrane integrity loss typical of necrosis). Apoptotic cells include both early and late apoptotic cells. Early apoptotic cells are typically Annexin V-positive and PI-negative, indicating phosphatidylserine externalization without membrane rupture. While, late apoptotic cells are positive for both Annexin V and PI, reflecting the advanced stage of apoptosis where membrane integrity is compromised. Necrotic cells are characterized by Annexin V-negative and PI-positive staining, indicating that their cell membranes are damaged and they are in the late stages of cell death (Rauf et al. 2024; Breckenridge and Xue 2004; Birkinshaw and Czabotar 2017; Mohamed et al. 2025c).

Additionally, the upregulation of the anti-apoptotic Bcl2 gene and the downregulation of the pro-apoptotic *p53* gene observed in the current study suggest that mitochondrial dysfunction plays a key role in the apoptotic process. The *Bcl2* family plays a crucial role in regulating mitochondrial outer membrane permeabilization, a key event in apoptosis. Paradoxically, overexpression of *Bcl2* can promote apoptosis by disrupting cellular homeostasis and interacting with other *Bcl2* family members, as well as through mechanisms that bypass *Bcl2*’s inhibitory effects. As a result, increased *Bcl2* expression significantly enhances cellular sensitivity to apoptosis, especially in the presence of mitochondrial stressors (Breckenridge and Xue 2004; Birkinshaw and Czabotar 2017; Mohamed et al. 2025c).

Mitochondrial dysfunction is a critical factor in cell death, and the disruption of mitochondrial DNA integrity is linked to various forms of cellular damage, including apoptosis (Khan et al. 2022). Induction of mitochondrial damage by Er_2_O_3_-NPs in U937 LCs was demonstrated by dramatic reduction in mitochondrial membrane potential and notable alteration in the expression of the mitochondrial *ND3* gene following 48 h of U937 exposure to Er_2_O_3_-NPs. These findings are consistent with recent research indicating that Er_2_O_3_-NPs can cause mitochondrial dysfunction through excessive ROS generation, leading to a breakdown in cellular energy metabolism and triggering apoptotic pathways (Safwat et al. 2022). Consequently, the imbalance in apoptotic gene expression observed after Er_2_O_3_-NPs treatment likely contributes to mitochondrial dysfunction, activating cell death pathways. This supports previous studies showing that ROS-induced mitochondrial damage leads to oxidative stress, mitochondrial dysfunction, and the release of apoptotic factors, ultimately driving apoptosis (Mohamed et al. 2025c; Qian et al. 2022).

In addition to ROS generation, mitochondrial dysfunction is another critical aspect of the cytotoxicity induced by Er_2_O_3_-NPs. Mitochondria are not only the powerhouses of the cell but also key regulators of cell death. The mitochondrial *ND3* gene, which encodes a subunit of the mitochondrial respiratory chain, was significantly downregulated in Er_2_O_3_-NPs-treated U937 LCs, further supporting the notion that these nanoparticles disrupt mitochondrial function. Mitochondrial damage is a known contributor to the activation of apoptosis, as impaired mitochondrial function leads to an increased release of pro-apoptotic factors and further ROS production (Mohamed et al. 2025c; Kuo et al. 2022).

The generation of ROS plays a crucial role in the mitochondrial membrane depolarization process. Regarding mitochondrial function, the accumulation of ROS can overwhelm the mitochondrial antioxidant defense mechanisms, leading to oxidative stress. This stress induces several critical events, including the disruption of the mitochondrial membrane potential. Over ROS generation can lead to the oxidation of key mitochondrial components, including mitochondrial DNA and proteins. This damage disrupts the mitochondrial electron transport chain, causing further ROS production. The increase in ROS levels can also trigger the opening of the mitochondrial permeability transition pore, a key event in mitochondrial dysfunction. This pore opening results in the loss of mitochondrial membrane potential, which is crucial for ATP synthesis and maintaining cellular energy balance. Moreover, the loss of mitochondrial membrane potential is a hallmark of the early stages of apoptosis. When the mitochondrial membrane depolarizes, it leads to the release of pro-apoptotic factors, such as cytochrome c, into the cytosol. This release activates caspases, which in turn drive the cell towards programmed cell death (Kumar et al. 2023, 2024c).

Moreover, genomic DNA damage, as observed in this study, is another critical outcome of nanoparticle exposure. The significant in DNA damage parameters: tail length, %DNA in tail and tail moment noticed in Er_2_O_3_-NPs-treated U937 LCs highlights the high induction of genomic DNA damage. ROS can directly damage DNA, leading to mutations, strand breaks, and other forms of genomic instability. The notable downregulation of the p53 gene, which plays a pivotal role in DNA damage response and cell cycle regulation, suggests that Er_2_O_3_-NPs may impair the cellular repair mechanisms and promote genomic instability. This finding is consistent with previous studies demonstrating that nanoparticles particularly Er_2_O_3_-NPs can induce genotoxic effects by generating ROS that damage the DNA (Mohamed et al. 2025a, c; Safwat et al. 2022).

## Conclusion

In summary, the results of this study demonstrate that Er_2_O_3_-NPs induce strong cytotoxicity in highly aggressive U937 LCs. This cytotoxicity is linked to the generation of excessive ROS, which causes substantial damage to both genomic and mitochondrial DNA. Additionally, the disruption of apoptotic, anti-apoptotic, and mitochondrial gene expression triggers apoptosis in U937 cells. These findings suggest that Er_2_O_3_-NPs show potential as a therapeutic agent for lymphoma. However, further in vivo studies, potential toxicity to normal cells, and optimization of the Er_2_O_3_-NPs for clinical applications are crucial to validate these findings and to better understand the full therapeutic potential of Er_2_O_3_-NPs. Moreover, future research is needed to optimize the design of Er_2_O_3_-NPs, improving their efficacy while minimizing toxicity to healthy cells for safe clinical application.

## Data Availability

The datasets used and/or analyzed during the current study are available from the corresponding author on reasonable request.
